# RAMPART: a workflow management system for *de novo* genome assembly

**DOI:** 10.1093/bioinformatics/btv056

**Published:** 2015-01-30

**Authors:** Daniel Mapleson, Nizar Drou, David Swarbreck

**Affiliations:** The Genome Analysis Centre, Norwich Research Park, Norwich NR4 7UH, UK

## Abstract

**Motivation:** The *de novo* assembly of genomes from whole- genome shotgun sequence data is a computationally intensive, multi-stage task and it is not known *a priori* which methods and parameter settings will produce optimal results. In current *de novo* assembly projects, a popular strategy involves trying many approaches, using different tools and settings, and then comparing and contrasting the results in order to select a final assembly for publication.

**Results:** Herein, we present RAMPART, a configurable workflow management system for *de novo* genome assembly, which helps the user identify combinations of third-party tools and settings that provide good results for their particular genome and sequenced reads. RAMPART is designed to exploit High performance computing environments, such as clusters and shared memory systems, where available.

**Availability and implementation:** RAMPART is available under the GPLv3 license at: https://github.com/TGAC/RAMPART.

**Contact:** daniel.mapleson@tgac.ac.uk

**Supplementary information:** Supplementary data are available at *Bioinformatics* online. In addition, the user manual is available online at: http://rampart.readthedocs.org/en/latest.

## 1 Introduction

The *de novo* genome assembly of whole genome sequence data is a complex task and typically involves testing multiple tools, parameters and approaches to produce the best possible assembly for downstream analysis. This is necessary because it is not always known *a priori*, which tools and settings will work best on the available sequence data given the organism’s specific genomic properties, such as size, ploidy and repetitive content. Despite advances in computing hardware and sequencing technologies, *de novo* assembly, particularly for more complex eukaryotic genomes, remains a non-trivial task and an ongoing challenge.

Recently, several tools, such as iMetAMOS (Koren *et* *al.*, 2014) and A5 (Tritt *et* *al.*, 2012), approach this problem by exhaustively testing many tools in parallel and then identifying and selecting the best assembly. However, these pipelines focus on prokaryote assemblies, where the computational demands are manageable and the genomes are easier to assemble. The complexities of eukaryotic genomes prohibit exhaustive testing of all tools and parameters with current computing hardware. For these projects the user must use the literature and their own experience to decide which avenues are worth considering.

## 2 RAMPART

This article presents a workflow management system for *de novo* genome assembly called RAMPART, which allows the user to design and execute their own assembly workflows using a set of third-party open-source tools. This reduces human error and relieves the burden of organizing data files and executing tools manually. Frequently, this helps to produce better assemblies in less time than is possible otherwise.

RAMPART gives the user the freedom to compare tools and parameters to identify the effect these have on the given data sets. The flexibility to roll-your-own workflow enables the user to tackle both prokaryotic and eukaryotic assembly projects, tailoring the amount of work to be done based on the availability of computing resources, quantity of sequence data and complexity of the genome. In addition, RAMPART produces logs, metrics and reports throughout the workflow, which allows users to identify, and subsequently rectify, any problems.

### 2.1 Workflow design

**Fig. 1.**
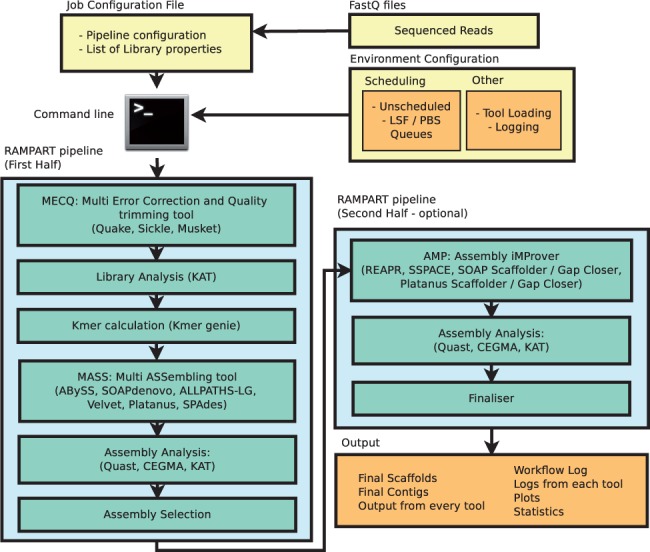
A simplified representation of RAMPART’s architecture. Although user’s workflow must conform to the linear structure depicted here, each stage is optional and highly configurable. Most stages allow the user to select which third-party tool(s) and parameters are used, although primary input and output parameters to all tools are managed automatically. The most important pipeline stage, MASS, allows the user to execute multiple assemblers, with varying parameters. In the subsequent step, the resultant assemblies are analyzed before a single assembly is selected for use in the second half of the pipeline. Input to the MASS and AMP stages can be selected from any raw input library or from any modified libraries generated during the MECQ stage

Input to RAMPART consists of one or more sequenced whole genome shotgun libraries and a configuration file describing properties of those libraries and the workflow through which the libraries should be processed. The workflow is comprised of a number of configurable stages as depicted in Figure 1. This design allows the user to answer project-specific questions such as: whether raw or error corrected sequence data works best; which assembler works best; or which parameter value is optimal for a specific tool. The final output from RAMPART is the assembled sequences, although plots, statistics, reports and log files are produced as the pipeline progresses.

RAMPART connects standardized input and outputs from adjacent pipeline stages, which in some cases requires translating in order to drive specific third-party tools. Designing the software this way has three advantages. First, the user only needs to install those tools required for their specific project. Second, the user does not have to manually specify many input and output parameters for the tools, particularly library properties and file locations. Finally, RAMPART developers can add new tools without changing the pipeline logic. RAMPART is an open source project so any user with the right skillset can add their own tools to their pipeline, providing those tools can be made to comply with appropriate interfaces.

### 2.2 Assembly comparison and selection

To compare assemblies, RAMPART measures properties of each assembly relating to contiguity, conservation and assembly problems using third-party tools. The user can control which analysis tools, if any, are executed in their pipeline. To function as a fully automated pipeline, RAMPART, at particular stages, must be capable of selecting the best assembly to proceed with. We address this by assigning a single score to each assembly using a method similar to that described by Abbas *et al*. (2014), which groups and weights individual assembly metrics before assigning a single score. The user has the option to override the default weightings for automatic selection, or can select an assembly manually at their discretion. Please see Supplementary Material Section 2 for more information.

### 2.3 High performance computing support

Experimenting with *de novo* assembly for large, complex genomes is a computationally intensive process. Therefore, RAMPART is designed to exploit high performance computing environments, such as clusters or shared memory machines, by executing tools in parallel where possible via the system’s job scheduler. However, RAMPART still runs on desktop and server machines sequentially with sufficient resources. RAMPART currently supports both the Platform Load Sharing Facility and Portable Batch System schedulers, with plans to support Sun Grid Engine in the future.

## 3 Concluding remarks

RAMPART is a workflow management system for *de novo* genome assembly that provides an effective means of producing quality prokaryotic and eukaryotic assemblies by reducing the amount of manual work required in such projects. In addition, it offers a way for users to better understand differences in their genomic sequence data, assemblies and assembly tools. RAMPART is already used in production workflows at The Genome Analysis Centre, is under active development and is updated regularly to adapt to the latest challenges, tools and data.

As sequencing costs have come down it has been possible to sequence multiple isolates of the same species in parallel, these kinds of projects present additional challenges for the bioinformatican in terms of managing the numbers of files and comparing results of *de novo* assemblies across isolates. RAMPART contains some preliminary scripts for managing these kinds of projects. It also enables the rapid functional annotation of prokaryote genomes via PROKKA (Seemann, 2014). In the future we would like to improve these scripts and workflows and to provide the ability to annotate eukaryotic genomes.

Over time, the community will develop a better understanding of what assembly workflows are appropriate for certain types of genomes with certain types of sequence data. For example, the ALLPATHS-LG ‘recipe’ (Gnerre *et* *al.*, 2011) has been shown to produce high-quality assemblies of mammalian genomes. We plan to encourage this process in the future by allowing users to share their own RAMPART workflows and metrics describing their results on a website for appraisal by the community.

## Supplementary Material

Supplementary Data
