# Supplementary material for: RAMPART: a workflow management system for de novo genome assembly
Source: Bioinformatics. 2015 Jan 30;31(11):1824–6. doi: 10.1093/bioinformatics/btv056 (PMC4443680; doi:10.1093/bioinformatics/btv056)
Supplement: Supplementary Data [file supp_31_11_1824__index.html]

RAMPART: a workflow management system for de novo genome assembly — RAMPART: a workflow management system for de novo genome assembly — Supplementary Data 

# RAMPART: a workflow management system for *de novo* genome assembly

## Supplementary Data

files

**Files in this Data Supplement:**

- Supplementary Data - pdf file
