# Supplementary material for: RAMPART: a workflow management system for de novo genome assembly
Source: Bioinformatics. 2015 Jan 30;31(11):1824–6. doi: 10.1093/bioinformatics/btv056 (PMC4443680; doi:10.1093/bioinformatics/btv056)
Supplement: Supplementary Data [file supp_btv056_supplementary.pdf]

RAMPART: Supplementary Material

Daniel Mapleson, Nizar Drou and David Swarbreck

January 5, 2015

1 Tools

RAMPART supports a number of third-party tools that can be combined into pipelines, allowing the user to define their own "recipes" for *de novo* genome assembly. Some tools are not initially compatible with each other, where this is the case RAMPART will convert outputs from one tool so they are compatible as input for another. For example, in the case where SOAP scaffolder is used, any contigs containing unknown nucleotides ('N') must be split at these positions first, RAMPART takes care of these kinds of issues so the user does not need to worry about them. The list of tools currently supported in RAMPART V0.11.0 are described in Table 1.

Tool Name	Type	Version	Reference
Sickle	RP	1.2	https://github.com/najoshi/sickle
Quake	RP	0.3	(Kelley *et al.*, 2010)
Musket	RP	1.0	(Liu *et al.*, 2013)
ABySS	ASM	1.5	(Simpson *et al.*, 2009)
ALLPATHS-LG	ASM	50***	(Gnerre *et al.*, 2011)
Platanus	ASM	1.2	(Kajitani *et al.*, 2014)
SOAP de novo	ASM	2.4	(Luo *et al.*, 2012)
SPAdes	ASM	3.1	(Bankevich *et al.*, 2012)
Velvet	ASM	1.2	(Zerbino and Birney, 2008)
Platanus scaffolder	AE	1.2	(Kajitani *et al.*, 2014)
Platanus gap closer	AE	1.2	(Kajitani *et al.*, 2014)
SOAP scaffolder	AE	2.4	(Luo *et al.*, 2012)
SOAP gap closer	AE	1.12	(Luo *et al.*, 2012)
SSPACE	AE	2.0	(Boetzer *et al.*, 2011)
REAPR	AE	1.0	(Hunt *et al.*, 2013)
Quast	AA	2.3	(Gurevich *et al.*, 2013)
CEGMA	AA	2.4	(Parra *et al.*, 2007)
KAT	AA	1.0	http://www.tgac.ac.uk/KAT
Kmer Genie	MISC	1.6	(Chikhi and Medvedev, 2013)
Subsampler	MISC	1.0	https://github.com/ homonecloco/subsampler

Table 1: Third-party tools supported by RAMPART V0.11.0. Tool type acronyms: RP - read processing; ASM = assembler; AE - assembly enhancer; AA - assembly analysis; MISC - miscellaneous

2 Assembly Selection

In order to assess the quality of each assembly, RAMPART combines results from several third-party assembly analysis tools. First, the majority of assembly metrics are gathered using Quast (Gurevich *et al.*, 2013).

Second, to gauge genome completeness for eukaryotic genomes, CEGMA (Parra *et al.*, 2007) is used. Finally, a k-mer based analysis is provided using KAT (http://www.tgac.ac.uk/KAT) that identifies what content present in the input is also present in the assembly. The user can control which, if any, of these assessment tools are executed in their pipeline. The specific set of assembly metrics used by RAMPART are defined in Table 2. The metrics are grouped according to their function (contiguity, problems, conservation) in a manner similar to that described in (Abbas *et al.*, 2014).

Metric	Group	From Tool	Description
# seqs	Contiguity	Quast	The number of assembled sequences in the assembly
# seqs > 1k	Contiguity	Quast	The number of assembled sequences with length greater than 1,000 bases
Largest seq	Contiguity	Quast	The longest sequence in the assembly
N50	Contiguity	Quast	The length for which the collection of all assembled sequences of that length or longer covers at least half the assembly
NA50	Contiguity	Quast	Requires reference. N50, when assembly is broken at missassembled points
L50	Contiguity	Quast	The number of sequences as long as N50
# bases	Conservation	Quast	The assembly size
# bases > 1k	Conservation	Quast	The assembly size from sequences with length greater than 1,000 bases
GC%	Conservation	Quast	Average GC% of the assembly
# genes	Conservation	Quast	The number of predicted genes in assembly
completeness	Conservation	CEGMA	The percentage of complete core eukaryotic genes found in all CEGMA groups
N%	Problem	Quast	Fraction of the assembly made up of unknown bases
# misasms in ref	Problem	Quast	Requires reference. The number of missasseblies found when comparing to the reference.
Contiguity Score	Group Score	RAMPART	A weighted average of the scaled sum of all contiguity metrics.
Conservation Score	Group Score	RAMPART	A weighted average of the scaled sum of all conservation metrics.
Problem Score	Group Score	RAMPART	A weighted average of the scaled sum of all problem metrics.
Final score	-	RAMPART	A weighted average of the scaled sum of the contiguity, problems and conservation scores.

Table 2: Metrics, ordered by group, used to assess each assembly in RAMPART. The raw metrics themselves are not calculated by RAMPART directly (except for the group and final scores), but are instead gathered from third-party tools (by Quast (Gurevich *et al.*, 2013) in particular).

The assembly scoring system works by first assigning a score for each metric group for each assembly, then a for final assembly score is derived from the group scores. Each group score, and the final score, is calculated from a weighted average derived from scaled metrics in their respective group. The scaling procedure for each metric results in a score of 0 representing the worst assembly for this metric and 1 being the best. The scaling procedure is derived from the standard feature rescaling equation $x' = \frac{x - \min X}{\max X - \min X}$. Note that feature rescaling means the score is only relevant for the given job, and therefore scores cannot be compared between jobs. The particular feature rescaling equation used for a given metric depends on the metric class:

- Metrics where higher values represent better assemblies
- Metrics where lower values represent better assemblies
- Metrics where values closer to a defined value represent better assemblies

To cater for these we must alter the standard feature rescaling equation so that each scaled value lays within the range of 0 and 1, where 0 implies the worst score for that particular metric and 1 implies the best score for that metric. More formally, first let m be a specific type of metric in the set of metrics M, and let a be an assembly from a set of assemblies A that are to be compared. r_{am} is then the raw value for metric m for assembly a. For each metric we scale the raw metric values r_{am} to a range between 0 and 1 s_{am}, using derivations of the standard feature rescaling equation to cater for the different metric types:

- When higher values are best: $s_{am} = \frac{r_{am} - \min(r_m)}{\max(r_m) - \min(r_m)}$
- When lower values are best: $s_{am} = 1 - \frac{r_{am} - \min(r_m)}{\max(r_m) - \min(r_m)}$
- When values closer to target are best: $s_{am} = 1 - \frac{|r_{am} - v_m|}{\max(|r_m - v_m|)}$

Where v_m is the known optimal value for the given metric m. For example v_m could refer to the estimated or known genome size.

Metrics belong to particular groups $g \in G$, where for each metric m there exists a group weighting w_m where $\sum_{m \in M_g} w_m = 1$. The score for each metric group for each assembly z_{ga} is calculated by summing the product of each scaled metric value s_{am} for that assembly by the weighting for that metric w_m:

$z_{ga} = \sum_{m \in M_g} s_{am} w_m$

The same weighting and summing process is then applied to each group score to calculate a single overall assembly score z_a. This allows the user to control the weightings at a higher level, which can be used to emphasise assembly quality over contiguity for example. The best assembly b is then determined to be the assembly with the highest final score: $z_b = \max z$. It is possible that $\max z$ may return more than one assembly. If this is the case, the first assembly in the set to have the highest score is selected.

For calculating assembly scores RAMPART specifies a default set of metric weightings, which we have found to be useful (Table 3). However, the user is free (and strongly encouraged) to provide their own metric weightings, or to disregard the assembly scores and select their own assemblies. It is important for users to make their own assembly assessment as the scoring system is biased by outlier assemblies. For example, consider three assemblies with an N50 of 1000, 1100 and 1200 bp, with scaled scores of 0, 0.5 and 1. We add a third assembly, which does poorly and is disregarded by the user, with an N50 of 200 bp. Now the weighted N50 scores of the assemblies are 0, 0.8, 0.9 and 1. Even though the user has no intention of using that poor assembly, the effective weight of the N50 metric of the three good assemblies has decreased drastically by a factor of (1 - 0) / (1 - 0.8) = 5. It's possible that the assembly selected as the best would change by adding an irrelevant assembly. For example consider two metrics, a and b, with even weights of 0.5 for three assemblies, and then again for four assemblies after adding a fourth irrelevant assembly, which performs worst in both metrics. By adding a fourth irrelevant assembly, the choice of the best assembly has changed.

Three assemblies:
$a = (1000, 1100, 1200), b = (0, 10, 8)$
$s_a = (0, 0.5, 1), sb = (0, 1, 0.8)$
$z_a = (0, 0.75, 0.9)$
$z_b = 0.9$

Four assemblies:
$a = (200, 1000, 1100, 1200), b = (0, 0, 10, 8)$
$s_a = (0, 0.8, 0.9, 1), sb = (0, 0, 1, 0.8)$
$z_a = (0, 0.4, 0.95, 0.9)$

$z_b = 0.95$

Note that for three assemblies the the assembly with $a = 1200$ is selected as best whereas for four assemblies the assembly with $a = 1100$ is selected as best. To reiterate, we recommend that the user verify the results provided by RAMPART and if necessary overrule the choice of assembly selected for further processing.

Metric	Type	Weighting
# seqs	Contiguity	0.1
# seqs > 1k	Contiguity	0.1
Largest seq	Contiguity	0.1
N50	Contiguity	0.3
NA50	Contiguity	0.3
L50	Contiguity	0.1
# bases	Conservation	0.1
# bases > 1k	Conservation	0.1
GC%	Conservation	0.1
# genes	Conservation	0.2
completeness	Conservation	0.5
N%	Problems	0.4
# misassemblies in ref	Problems	0.6
Contiguity	Group Scores	0.5
Problems	Group Scores	0.2
Conservation	Group Scores	0.3

Table 3: The default weightings for metrics in RAMPART V0.11.0, divided into each metric group. The weightings for each group should sum to 1.0 in order to return scores between 0.0 and 1.0. The final set of metrics (contiguity, problems, conservation) are scores which are derived from the groups above, and are then used to calculate the final score for each assembly. These weightings may change in future RAMPART versions.

3 Runtimes and Resource Usage

The following statistics are provided only in order to provide an idea of relative runtimes and memory usage of each tool. The purpose is to help the user construct pipelines that are suitable for their own computing systems. The purpose is not to make any comment on the relative efficiencies of the tools. These figures are based on the current versions used in RAMPART at the time of publication and would be subject to change when newer versions are released, or used on different systems.

Actual runtimes may vary on the user's system relative to the computing power available. Tools were run on AMD Opteron848 CPUs with 128GB RAM connected to an Isilon network attached storage system. The assembly project was for a diploid diatom genome of approximately 69MB. The input datasets used are described in Table 4. In addition, where required a contig assembly was used. The assembly contained 202,917 sequences, totalling 75,065,477 bp in length. The N50 was 6,385 bp. Runtimes for each tool are shown in Table 5.

To give an impression of how long typical assembly projects may take we provide details in table 6. The table indicates approximate end-to-end resource requirements and runtimes for a set of different genomes. Users experience may vary depending on size of input files, specific genome properties and computing hardware capabilities.

ID	Nb reads	Length	Mean Insert Size	Depth (approx)
OPE	29,342,483	101bp (all)	180bp	86X
PE	29,252,071	100bp (all)	450bp	84X
MP	5,387,728	156bp (mean)	4,500bp	24X

Table 4: Datasets used for runtime tests. PE is sequenced from an illumina paired end library. OPE is an illumina sequenced overlapping paired end library. MP is sequenced from an illumina nextera mate pair library. Reads in this library were originally 300bp long before processing by nextclip (Leggett *et al.*, 2014) to remove adaptors and retain good mate-pairs (category A,B and C reads were retained).

Tool	OPE	PE	MP	ASM	Threads	MaxMem(MB)	Time(s)	CPUTime(s)
Sickle		✓			1	3	336	308
Quake		✓			32	16,740	2,010	8,925
Musket		✓			32	2,592	1,859	52,004
ABySS		✓			32	16,637	5,426	253,232
ALLPATHS-LG	✓		✓		32	75,905	40,101	614,764
Platanus ASM		✓			32	24,633	1,552	22,413
SOAP ASM		✓			32	49,421	1,055	11,901
SPAdes		✓			32	19,117	14,953	121,515
Velvet		✓			32	19,975	1,493	12,171
Platanus Scaff			✓	✓	32	3,767	352	6,353
Platanus GC		✓		✓	32	2,784	303	1,131
SOAP Scaff			✓	✓	32	9,735	319	2,121
SOAP GC		✓		✓	32	7,881	1,695	38,931
SSPACE			✓	✓	32	1,097	409	5,463
Quast				✓	32	28	14	14
CEGMA				✓	32	1,906	6,381	26,320
KAT		✓		✓	32	3,387	396	3,143
REAPR		✓		✓	32	1,833	16,507	91,765
Kmer Genie		✓			32	925	3,361	22,727
Subsampler		✓			1	2	454	349

Table 5: Runtimes and memory requirements for all tools in RAMPART on the same datasets.

Genome	Genome Size (Mbp)	Ploidy	MaxMem (GB)	Time (hrs)	CPUTime (hrs)
Escherichia coli	4	1	23	3	52
Arabidopsis thaliana	135	2	67	35	369
Apis cerana	250	2	154	112	785

Table 6: Approximate runtimes and memory requirements for various types of genome projects. Same hardware was used for each project although actual workflows and tools used differ to make them appropriate for the available data and genome properties.

References

Abbas, M.M., Malluhi, Q.M. and Balakrishnan, P. (2014). Assessment of de novo assemblers for draft genomes: a case study with fungal genomes. *BMC Genomics*, **15 Suppl 9**, S10.

Bankevich, A. et al (2012). Spades: a new genome assembly algorithm and its applications to single-cell sequencing. *J Comput Biol*, **19**(5), 455–477.

Boetzer, M. et al (2011). Scaffolding pre-assembled contigs using SSPACE. *Bioinformatics*, **27**(4), 578–579.

Chikhi, R. and Medvedev, P. (2013). Informed and automated k-mer size selection for genome assembly. *Bioinformatics*.

Gnerre, S. et al (2011). High-quality draft assemblies of mammalian genomes from massively parallel sequence data. *Proceedings of the National Academy of Sciences*, **108**(4), 1513–1518.

Gurevich, A. et al (2013). QUAST: quality assessment tool for genome assemblies. *Bioinformatics*, **29**(8), 1072–1075.

Hunt, M. et al (2013). REAPR: a universal tool for genome assembly evaluation. *Genome Biol*, **14**(5), R47.

Kajitani, R. et al (2014). Efficient de novo assembly of highly heterozygous genomes from whole-genome shotgun short reads. *Genome Res.*, **24**(8), 1384–1395.

Kelley, D.R., Schatz, M.C. and Salzberg, S.L. (2010). Quake: quality-aware detection and correction of sequencing errors. *Genome Biology*, **11**(11), R116.

Leggett, R.M. et al (2014). Nextclip: an analysis and read preparation tool for nextera long mate pair libraries. *Bioinformatics*, **30**(4), 566–568.

Liu, Y., Schroder, J. and Schmidt, B. (2013). Musket: a multistage k-mer spectrum-based error corrector for illumina sequence data. *Bioinformatics*, **29**(3), 308–315.

Luo, R. et al (2012). SOAPdenovo2: an empirically improved memory-efficient short-read de novo assembler. *GigaScience*, **1**(1), 18.

Parra, G., Bradnam, K. and Korf, I. (2007). CEGMA: a pipeline to accurately annotate core genes in eukaryotic genomes. *Bioinformatics*, **23**(9), 1061–1067.

Simpson, J.T. et al (2009). ABySS: a parallel assembler for short read sequence data. *Genome Res*, **19**(6), 1117–1123.

Zerbino, D.R. and Birney, E. (2008). Velvet: algorithms for de novo short read assembly using de bruijn graphs. *Genome Res*, **18**(5), 821–829.
